# A Case of Docetaxel Induced Myositis and Review of the Literature

**DOI:** 10.1155/2015/795242

**Published:** 2015-07-16

**Authors:** Alexandra Perel-Winkler, Regina Belokovskaya, Isabelle Amigues, Melissa Larusso, Nazia Hussain

**Affiliations:** ^1^Department of Medicine, St. Luke's-Roosevelt Hospital Center, and Department of Medicine, Icahn School of Medicine at Mount Sinai, New York, NY 10025, USA; ^2^Division of Rheumatology, Department of Medicine, St. Luke's-Roosevelt Hospital Center, and Department of Medicine, Icahn School of Medicine at Mount Sinai, New York, NY 10025, USA

## Abstract

In phase I and II trials taxane chemotherapeutic agents reported side effects, including myelosuppression, peripheral edema, and fluid retention. With further use of these agents, studies in the late 1980s and early 1990s began to report peripheral neuropathy and proximal muscle weakness as common complaints, the later with unexplained pathophysiology. We report a 65-year-old Hispanic woman with estrogen receptor (ER) and progesterone receptor (PR) positive invasive ductal breast carcinoma who presented with right thigh pain and swelling eight days after her third infusion of docetaxel (a taxane chemotherapeutic) and cyclophosphamide. Laboratory findings were notable for elevation in creatine phosphokinase (CPK), aldolase, and erythrocyte sedimentation rate (ESR); a magnetic resonance imaging (MRI) of her lower extremities showed evidence of bilateral muscle edema involving the anterior compartment muscles of the thighs. A workup to rule out other causes of myositis was negative. Docetaxel was not reintroduced and the patient improved with corticosteroids. Since 2005 this is, to our knowledge, the fifth reported case of docetaxel related inflammatory myositis. Taxanes have been noted to cause disabling but transient arthralgias and myalgias; it is important to consider the possibility of inflammatory myopathy as a possible complication in patients undergoing treatment with these agents.

## 1. Introduction

Taxane drugs, paclitaxel and docetaxel, are chemotherapeutic agents which work by disrupting microtubule function to inhibit cell division. Docetaxel has become a frequently used agent, known for its efficacy in solid tumors, primarily breast cancer, metastatic prostate cancer, and non-small cell lung cancer. Breast cancer is the most common cancer and the second leading cause of cancer death in American women [1]. Medical advances have considerably improved survival, largely due to newer anticancer medications. With efficiency, these agents also come with new side effects which physicians should be made aware of. Both paclitaxel and docetaxel (the taxanes) are known to cause myalgias, arthralgias, and neuropathy; however, there are few studies describing direct muscle inflammation caused by these agents. Myositis, an inflammation of muscle, can be caused by injury, infection, medications, toxins, exercise, or autoimmune disease. In this report we describe the case of a patient who developed a case of docetaxel induced myositis when undergoing treatment for invasive ductal breast cancer. We will also include a description of other inflammatory muscle reactions in the setting of taxane agents.

## 2. Case

A 65-year-old Hispanic female presented to our Emergency Department with one week of right thigh pain and swelling. Her past medical history includes asthma, peripheral vascular disease, coronary artery disease, seizure disorder, hypertension, and hyperlipidemia. She was diagnosed with poorly differentiated invasive ductal carcinoma, with estrogen and progesterone receptor (ER and PR) positivity and human epidermal growth receptor 2 (HER2) negative, of her right breast in July 2013. She underwent a right breast lumpectomy and was started on adjuvant chemotherapy with a plan for 4 cycles of docetaxel 75 mg/m^2^ IV and cyclophosphamide 600 mg/m^2^ IV every 3 weeks. She completed 3 cycles and had tolerated the treatment well with no notable side effects, until she presented to the ED eight days after the last infusion complaining of right thigh pain. Her medications at the time of presentation are listed in Table 1. No changes had been made to any of her medications in the last few months prior to this presentation.

The patient had begun to develop rapidly progressive pain and swelling of her right thigh, without complaints of weakness, eight days after her third infusion of docetaxel and cyclophosphamide. On examination, she was hemodynamically stable and afebrile. Her right thigh was erythematous and tender to light touch. She had decreased active range of motion of her right hip. There were no skin lesions. Her neurological examination was unremarkable; she had neither objective muscle weakness nor sensory deficit and had normal deep tendon reflexes. There was no fasciculation or muscle wasting. Her distal pulses were palpable.

Basic laboratory values demonstrated a leukocytosis of 12 × 10^9^/L. There were no electrolyte abnormalities. Her creatinine phosphokinase (CPK) was elevated to 341 (normal range: 30–135 U/L), her aldolase was 13.3 (normal range: ≤  8.1 U/L), and her erythrocyte sedimentation rate (ESR) was 38 (normal range: 0–24 mm/hr). Autoantibodies including antinuclear antibody, double stranded DNA, anti-Smith, rheumatoid factor, cyclic citrullinated peptide, anti-Ro (SSA) and anti-La (SSB), ribonucleoprotein, and Scl 70 were all negative.

**Figure 1:**
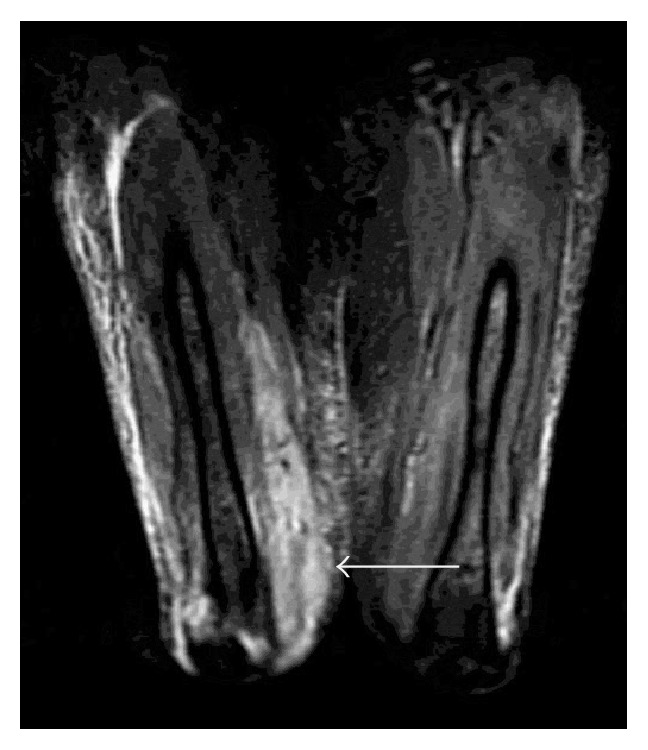
Axial T2-weighted FAT SAT image illustrating diffuse muscle edema involving the lower two-thirds of the anterior compartment muscles.

**Figure 2:**
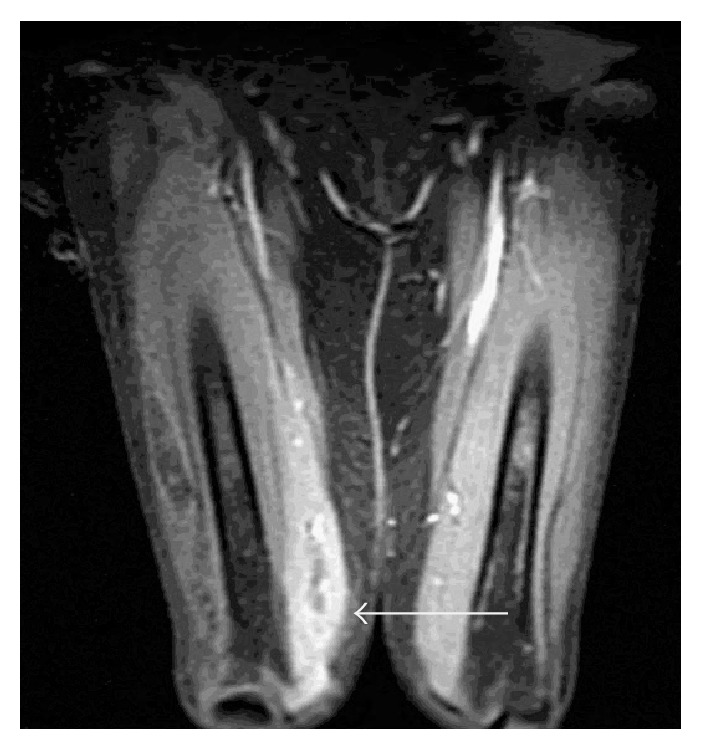
Coronal T2-weighted FAT SAT showing diffuse muscle edema involving the lower two-thirds of the anterior compartment muscles. The left thigh also shows muscle edema especially along the superior aspect of the rectus femoris and inferior aspect of the posterior compartment muscles.

Initially, the unilateral thigh pain and swelling in a patient undergoing chemotherapy and on oral steroids raised suspicion for cellulitis. The patient was commenced on a 7-day treatment of IV Vancomycin and Cefepime and transitioned to oral Clindamycin and Augmentin after the first week of antibiotics. The patient's right thigh pain did not improve while on antibiotics. An MRI of the thighs was done (Figures 1 and 2), which demonstrated diffuse muscle edema involving the lower two-thirds of the anterior compartment muscles. There was also patchy edema of the posterior compartment muscles especially at the level of the mid belly of the semitendinosus. On postgadolinium injection images, there were patchy areas of nonenhancement in some of the anterior compartment muscles. Small patch of nonenhancement was also noted in the long head of the rectus femoris. Subcutaneous edematous changes were noted especially along the lateral aspect of the thigh. Partially visualized left thigh images also showed muscle edema along the superior aspect of the rectus femoris and inferior aspect of the posterior compartment muscles. Gadolinium injection enhancement images were suggestive of myonecrosis. The overall findings were consistent with nonspecific myositis.

Upon review of her diagnostic results, recent medications, and chemotherapy treatments, it was suspected that docetaxel was the offending agent causing the myositis. Antibiotics were discontinued and the patient was commenced on prednisone 20 mg orally for 9 days and tapered down to 10 mg orally for another 5 days. The patient's symptoms improved significantly, including the erythema, tenderness, swelling, and range of motion. Her CPK normalized the day after prednisone was initiated.

Eight months after the last docetaxel treatment the patient remains asymptomatic without evidence of recurrent myositis. After her discharge the patient was treated with 4 months of radiation therapy. The patient is currently being treated with an aromatase inhibitor.

## 3. Discussion

Our case describes a patient with ER and PR + invasive ductal carcinoma presenting with unilateral thigh pain and swelling after her third cycle of docetaxel. Due to the asymmetric presentation the patient was initially treated for cellulitis without improvement. Further evaluation by MRI showed evidence of bilateral myositis, despite the unilateral symptoms. The MRI results alongside elevated CPK levels suggested acute myositis. The patient improved with a prednisone taper and symptoms did not recur after cessation of docetaxel treatment. It is important to note the rapid resolution of CPK, which is unusual for myositis. In this case, the presumed offending agent, docetaxel, had been held for almost two weeks at the point of diagnosis and an element of spontaneous resolution is to be expected.

Docetaxel is a taxane chemotherapeutic agent, which promotes the polymerization and inhibits depolymerization of microtubules causing interference of cell division. Docetaxel has been used with efficacy, usually in combination with another chemotherapeutic agent, in breast, ovarian, refractory prostate, head and neck, gastric, and non-small cell lung cancers [10]. The most commonly recorded side effects of taxane agents are peripheral edema and fluid retention; however, the drug can cause dose dependent, severe myelosuppression, most commonly neutropenia. Myalgia and neuropathies were not noted as common taxane side effects in the early trials; however, studies in the late 1980s to mid-1990s began reporting frequent peripheral neuropathy [11–13] and myalgias [14–18] as side effects in patients receiving docetaxel. Neuropathy, paresthesia, myalgias, and arthralgias are now known to be common complications of these chemotherapeutic agents, recognized mostly by clinicians due to patient discomfort. These complaints, however, have not been considered significant by the research community, as they are not an indication for cessation of treatment. More recent research has shown that, despite the description of these side effects as “not significant” in most of phase II and subsequent trials, clinical experience and recent evidence are showing that up to 79% of patients develop toxicity leading to pain during treatment with taxanes [19]. While myositis has not been commonly attributed to these drugs, it is an important consideration in light of the large degree of patient who reported myalgias, weakness, and pain.

While Lipton et al. were the first to report taxane induced neuropathy in 1989 [20], in 1996 Freilich et al. described evidence of proximal muscle weakness in patients participating in phase II docetaxel trials. In this study 60 patients were prospectively followed as they were treated with docetaxel (fifty-four) or paclitaxel (six). The authors evaluated neurologic complications. Seven patients in the docetaxel group developed weakness, graded as “mild objective weakness without significant impairment of function.” Other neurologic side effects were also reported including impairment of cutaneous sensation (two) and diminished reflexes (two). Interestingly, in 40% of patients treated with docetaxel who did not develop weakness, proximal myalgia was reported. In all patients, weakness abated after 1 to 2 months of drug cessation. The authors hypothesized that docetaxel may cause an idiosyncratic weakness that may occur at any stage of treatment and is associated with a predominantly proximal myopathy. The mechanism of the described proximal muscle weakness was unclear. Electrodiagnostic studies revealed a range of abnormalities including axonal sensorimotor neuropathy, multilevel radiculopathies, and absent lower limb motor and sensory responses. Of note, all patients had a reported CPK in normal range, suggesting a neuropathic etiology of weakness [21].

In 2005, two reports documented cases of acute inflammatory myositis developing in patients treated with docetaxel. Ardavanis et al. described the case of a 57-year-old man treated with a combination of gemcitabine and docetaxel for non-small cell lung cancer with positive results. After his fourth cycle of treatment the patient developed a symmetric proximal muscle weakness with elevations of CPK, lactate dehydrogenase (LDH), and aldolase. EMG and MRI investigation was not pursued due to the classic clinical picture and supportive lab results. The patient was treated with methylprednisolone and had resolution of muscle symptoms and normalization of enzymes within four weeks [2].

Hughes and Stuart-Harris described another case of docetaxel associated myositis in a 47-year-old woman with ER and PR + metastatic breast carcinoma. The patient experienced bilateral foot pain during her first cycle of treatment, which resolved between treatment cycles, and recurred after her second cycle with spontaneous resolution. After her third cycle of treatment the patient experienced a recurrence of her bilateral pain associated with weakness of her proximal lower extremities. The weakness progressed over 10 days to the point of difficulty with ambulation and transferring. The patient was found to have a markedly elevated CPK and was started on dexamethasone. After 6 days of treatment the patient was discharged home with strength sufficient to mobilize safely. Again, in this case, the clinical features and elevated CPK were taken as sufficient evidence of myositis and EMG; MRI and muscle biopsy were not performed [3].

Two other cases of docetaxel induced myositis were documented in separate clinical trials in 2006. Myositis was listed as a toxic side effect without details of the specific cases or description of workup for the diagnosis. Kalmadi et al. reported results from a phase II trial using docetaxel and gemcitabine as first-line therapy for non-small cell lung cancer. Of the 49 patients who were treated with this regimen one patient was noted to require dose adjustment for myositis [6]. Fardet et al. described the use of docetaxel and paclitaxel for treatment of non-HIV related Kaposi sarcoma. Twelve patients were enrolled in this study and one patient experienced “diffuse myalgia with biologic myositis” after treatment with a taxane agent; however, the specific agent was not specified [5].

In 2014, Winkelmann et al. described a case of a 64-year-old woman who was treated with paclitaxel and carboplatin for metastatic, poorly differentiated, serous adenocarcinoma. Between her second and third treatments the patient developed diffuse muscular weakness, as well as Raynaud's phenomenon, skin tightness, and gastroesophageal reflux. Further investigation showed an elevation of ESR, liver function tests (LFTs), and a CPK of 1523 U/L. While her serum Scl70, ANA, and anticentromere were negative, a punch biopsy showed thickened collagen bundles consistent with sclerosis. After completion of chemotherapy and treatment with methotrexate and prednisone, the patient's sclerosis improved and CPK trended downwards; however, muscle weakness persisted. A muscle biopsy was only done at this time, which showed nonspecific inflammation. This case exhibits two rare taxane induced symptoms, sclerosis and myositis [8].

Other interesting cases linking polymyositis with taxane agents have been described by Sasaki et al. and Gidron et al. (see Table 2). In the case described by Sasaki's group, a 58-year-old man with a type B2 thymoma, unrelated to Myasthenia Gravis (MG), was treated with 2 cycles of carboplatin and paclitaxel. Eighteen days after this treatment the patient was admitted to the hospital with fevers, chills, and muscle weakness. The patient was found to have elevation of LFTs and a CPK of 7271 U/L. While polymyositis and myocarditis have been associated with thymomas, these tumors are exceptionally rare and usually in association with MG [7]. Gidron et al. described a similar case of a 32-year-old woman with hairy cell leukemia and a large malignant thymoma, also treated with carboplatin and paclitaxel, after which she developed terminal polymyositis and myocarditis [4].

Perel-Winkler and Derk describe another case of diffuse cutaneous mucinosis and dermatomyositis in a 57-year-old man who was treated with paclitaxel and carboplatin for non-small cell lung cancer. The patient had good response to the treatment but developed a progressive, erythematous, pruritic rash four months after completion of his treatment. No active cancer was found on full body PET-CT; muscle enzymes were normal; however, MRI showed hyperenhancement of quadriceps muscles bilaterally. The patient did not respond to steroids or methotrexate and required IVIG for symptomatic resolution [9].

In our case and in many of the other published cases of docetaxel and paclitaxel induced myopathic toxicity (Table 2), there is no biopsy to support the diagnosis of myositis. Temporal relationship with recent use of the chemotherapeutic agents, alongside the laboratory findings of elevated muscle enzymes and supportive imaging, was accepted as sufficient to make a diagnosis. For academic purposes a biopsy is preferable to definitively rule out all other differentials. Below we describe potential differential diagnoses and discuss why we feel docetaxel induced myositis is the best diagnosis for our case.

In the setting of active malignancy, dermatomyositis (DM) and polymyositis (PM) should be considered in a patient presenting with muscle weakness, pain, and evidence of inflammation. Our patient did not have any of the classic cutaneous signs of DM, such as heliotropic rash, Gottron's papules, shawl sign, and/or erythematous plaques. Furthermore, in both DM and PM, the presentation of muscle pathology usually occurs earlier in the course of the malignancy and improves with successful treatment [22, 23]. Hence, we feel secure in associating the presence of myositis with the taxane agent and not as a sequela of the cancer itself.

Sensorimotor polyneuropathy can occur as a paraneoplastic syndrome or as a complication of chemotherapy treatments, including taxane agents as described above. With cases associated as side effects of treatment, the provider will likely note a progressive time course to presentation of symptoms. In the case of a paraneoplastic syndrome, neuropathic symptoms more often precede detection of malignancy. Paraneoplastic neurologic symptoms have varied presentations, including motor, sensory, and autonomic changes; muscle enzymes will remain in normal range and swelling is not a common presenting complaint [24].

Another rare cause of muscle pain and swelling which should be considered in an immunocompromised host is pyomyositis. In this pathology, a deep muscular infection exists and signs of infection are present including fevers and leukocytosis. On imaging one may note obvious abscesses within the thigh and gluteal muscles. Bacterial pyomyositis has been reported in association with both hematologic and solid organ malignancies. There have also been reports of toxicity-related pyomyositis in the setting of taxane agents. Two such cases have been described in the setting of paclitaxel treatment for endometrial cancer. Both patients presented classically with fever, pain, and decreased range of motion, but due to the rarity of the diagnosis appropriate treatment including drainage of the abscess collection was delayed [25, 26]. Our patient did not present with fever or leukocytosis; there was no evidence of abscesses which were noted on MRI and did not improve with antibiotics.

Diabetes can also cause both neurologic and myopathic complications. Diabetic lumbosacral radiculoplexus neuropathy (DLRPN), also known as diabetic amyotrophy, is a rare complication of diabetes causing a debilitating proximal diabetic neuropathy leading to weakness and pain of the pelvic girdle and proximal muscles of the lower extremities. DLRPN affects less than 1% of diabetic patients, and the risk of developing this disorder is unrelated to glycemic control. The first presentation of this syndrome is usually unilateral thigh pain leading to weakness and atrophy and may progress to bilateral and more distal weakness. Autonomic involvement may occur leading to bladder, bowel, and sexual disorder; sensory involvement is also reported in advanced cases. DLRPN is diagnosed by EMG and is often associated with nonspecific markers of inflammation and immune mediated disease markers such as a positive antinuclear antibody test or rheumatoid factor [27]. EMG results are usually suggestive of axonal degeneration. Biopsy reports show varied evidence of ischemic injury to nerves. The leading accepted theory of the pathophysiology of DLRPN is an immune mediated microvasculitis affecting the lumbar plexus. This disorder has an insidious presentation and is self-limiting; however, symptoms often persist from months to years [28]. In DLRPN patients often report symptoms in association with weight loss and good glycemic control and are not usually treated with insulin. In contrast, our patient's diabetic control was poor, with an HBA1c of 9.7%, and she was treated with insulin. The elevation in CPK and aldolase in our patient supports our diagnosis of myositis, whereas DLRPN's pathology is accepted as microvascular ischemia affecting nerves. Finally, our patient's quick recovery, within weeks, is not in line with the time course associated with DLRPN.

Diabetic myonecrosis must also be considered as a differential. This disorder is a rare complication of poorly controlled diabetes, usually occurring in patients with preexisting microvascular disease. Acute onset of pain and swelling of the thigh is the most common presenting complaint [29]. Laboratory workup in these patients is usually nondiagnostic, with normal white cell count and CPK, and moderately elevated ESR. Biopsies have been done, demonstrating muscle necrosis and edema; however, studies have shown that time to resolution doubled from 29 to 60 days after biopsy was performed. Biopsy, therefore, is not recommended for diagnosis. The modality of choice for further evaluation is MRI, which is claimed to be both sensitive and specific enough for diagnosis, although its specificity has been refuted [30]. Typical MRI features of diabetic myonecrosis include a hyperintense signal on T2-weighted images and an isointense to hypointense signal on T1-weighted images from the affected muscle, with associated marked edema and enhancement around irregular regions of muscle necrosis [31]. Our patient fits into the criteria of a poorly controlled diabetic, presenting with acute onset of thigh pain and swelling. The MRI images in our patient are more consistent with a diagnosis of an inflammatory myopathy, particularly the diffuse muscle edema and subcutaneous involvement. While diabetic myonecrosis cannot be completely ruled out without biopsy, the temporal relationship to docetaxel in our case makes this a more likely diagnosis.

Finally, drug-induced myopathy should also be considered in all cases of unexplained myalgias and weakness. Muscle toxicity can occur in association with many drugs. The large mass of muscle and its exposure to large amounts of blood flow make muscle a common source of adverse drug reactions. Drug-induced myopathy can be the cause of direct myotoxicity, most often associated with statins, colchicine, steroids, and antiretroviral agents [32]. Other mechanisms of drug-induced myopathy include inflammation, interference of neuromuscular transmission, or indirect metabolic causes, such as drug-induced hypokalemia, hypermagnesemia, or drug-induced hyperkinetic states [33].

Statin induced myopathy is a spectrum of disorders ranging from myalgias (muscle pain without CPK elevation) to myositis (muscle inflammation evidenced by CPK elevation) and rhabdomyolysis (debilitating muscle weakness with CPK > 10 times the normal range, often accompanied by kidney injury). While pain and muscle weakness are reported in 1–20% of patients taking statins, myositis and rhabdomyolysis reactions are rare and have been reported in less than 0.001% cases. Reactions to statins are related to dose but not time course, and drug-drug interactions are known to significantly affect the incidence of statin induced myopathies [34]. Most often drugs that increase the metabolism and systemic exposure of statins are implicated, such as gemfibrozil, nicotinic acid, macrolide antibiotics, azole antifungal agents, protease inhibitors, ranolazine, and calcium channel blockers [35]. Muscle related symptoms have been reported in simvastatin and atorvastatin more than other agents; however, severe rhabdomyolysis and myositis have not been reported in any agent more than another currently on the market. Biopsies of patients currently taking, or recently discontinued, statins with myopathy show extensive intracellular vacuolization in skeletal muscles. Visualization with electron microscopy indicated that intracellular vacuoles corresponded to membranous cavities in a distribution consistent with the T tubule system [36]. Our patient was taking a low dose of rosuvastatin concomitantly with a calcium channel blocker putting her at risk for statin associated myopathy. The statin and antihypertensive were stopped during her hospitalization and were not restarted as an outpatient. While statin induced myopathies can occur at any time, the temporal relationship with docetaxel is in line with the other reported cases of docetaxel induced myositis, favoring the later diagnosis. Further, amlodipine has been recognized to more often potentiate the myopathic toxicity of atorvastatin not rosuvastatin. Our diagnosis would be more convincing if there was a biopsy that differed from the findings of a statin induced myositis biopsy; however, as none of the reported cases of docetaxel induced myositis have a biopsy reported, there would not be strong comparison even if our patient had one.

In our case a docetaxel induced inflammatory myositis was the best diagnosis, in consideration of all the differentials. Diabetic myonecrosis and statin induced myopathy cannot be definitely ruled out without further investigation with muscle biopsy. We believe that, in comparing our case to another docetaxel related myositis and in consideration of the temporal relationship, it is highly likely that our patient's myositis was related to the taxane agent. Taxanes are known to cause disabling but transient arthralgias and myalgias in up to 75% of patients; these events typically occur 1 to 3 days after therapy and may significantly affect a patient's quality of life for several days [37]. It is important for practitioners to recognize that inflammatory myopathy is also a possible complication for patients when treated with these agents.

## 4. Conclusion

Neuromuscular side effects such as myalgia and neuropathy are now considered commonly known consequences of docetaxel treatment. Our case is the fifth documented case of inflammatory myositis in the setting of docetaxel treatment. A few other cases of myositis have been reported in association with the taxane agent, paclitaxel. Due to the small sample size, correlations between lengths of treatments, types of malignancy, or patient demographics cannot be made. It is important for clinicians to be aware that inflammatory myositis can be an adverse effect of docetaxel treatment. Myositis should be considered in the differential when managing a patient with muscle weakness after treatment with a taxane agent.

## Figures and Tables

**Table 1:** Home medications.

Medication	Dose
Amlodipine besylate	5 mg daily
Aspirin	81 mg daily
Nebivalol	5 mg daily
Dexamethasone	4 mg (2 tablets BID, only for 3 days starting 1 day before chemotherapy)
Docusate sodium	100 mg daily PRN
Ferrous sulfate	325 mg BID
Folic acid	1 mg daily
Hydrochlorothiazide	12.5 mg daily
Hydrocodone-acetaminophen	5–500 mg daily PRN
Levetiracetam	750 mg q 12 hours
Insulin glargine	45 units at bedtime
Linaclotide	145 mcg daily
Pregabalin	25 mg daily
Meclizine	12.5 mg 2 tablets daily
Memantine HCl	5 mg daily
Metoclopramide	10 mg q 6 hours PRN
Pegfilgrastim	6 mg once SubQ per chemotherapy cycle, beginning 24–72 hours after completion of chemotherapy
Esomeprazole	40 mg daily
Insulin aspart	12 units TIDAC
Ondansetron	8 mg TID for 2 days after chemotherapy
Prasugrel	5 mg daily
Ranitidine	150 mg daily
Rosuvastatin	10 mg daily
Trazodone	50 mg at bedtime
Vitamin C	500 mg 2 tabs. daily

**Table 2:** Documented case reports of taxane related myopathies.

Case	Demographics	Taxane ± other agents	Cancer type	Onset of muscle pathology	Treatment	Effect
Ardavanis et al. 2005 [2]	57, male	Docetaxel, gemcitabine	NSCLC	Day 7 after 4th cycle	Prednisone	Myositis of bilateral thighs

Hughes and Stuart-Harris, 2005 [3]	47, female	Docetaxel, epirubicin, and cyclophosphamide	Breast cancer, ER/PR+, HER2−	Day 11 after 2nd cycle	Prednisone	Myositis of bilateral thighs

Gidron et al., 2006 [4]	32, female	Paclitaxel, carboplatin	Hairy cell leukemia and thymoma	Day 7 after second cycle	IV corticosteroids, IVIG	Polymyositis and myocarditis (terminal)

Fardet et al., 2006 [5]	Unknown	Docetaxel or paclitaxel, agent unknown	Kaposi sarcoma	Unknown	Unknown	Unknown

Kalmadi et al., 2006 [6]	Unknown	Docetaxel and gemcitabine	NSCLC	Unknown	Unknown	Unknown

Sasaki et al., 2012 [7]	58, male	Paclitaxel, carboplatin	B cell thymoma	Day 18 after 2nd cycle	Not reported	Polymyositis, myocarditis (terminal)

Winkelmann et al., 2014 [8]	64, female	Paclitaxel, carboplatin	Ovarian adenocarcinoma	After second cycle	Methotrexate, prednisone	Polymyositis, scleroderma, Raynaud's and GERD

Perel-Winkler and Derk, 2014 [9]	57, male	Paclitaxel and carboplatin	NSCLC	Months	Prednisone, plaquenil, methotrexate, and IVIG	Mucinous dermatomyositis

Current case: Perel-Winkler et al.	65, female	Docetaxel and cyclophosphamide	Breast cancer (ER/PR+, HER2−)	Day 8 after 3rd cycle	Prednisone	Bilateral proximal thigh myositis R > L

## References

[1] American Cancer Society (2014). *Cancer Facts and Figures 2014*.

[2] Ardavanis A. S., Ioannidis G. N., Rigatos G. A. (2005). Acute myopathy in a patient with lung adenocarcinoma treated with gemcitabine and docetaxel. *Anticancer Research*.

[3] Hughes B. G. M., Stuart-Harris R. (2005). Docetaxel-induced myositis: report of a novel side-effect. *Internal Medicine Journal*.

[4] Gidron A., Quadrini M., Dimov N., Argiris A. (2006). Malignant thymoma associated with fatal myocarditis and polymyositis in a 32-year-old woman with a history of hairy cell leukemia. *American Journal of Clinical Oncology*.

[5] Fardet L., Stoebner P.-E., Bachelez H. (2006). Treatment with taxanes of refractory or life-threatening Kaposi sarcoma not associated with human immunodeficiency virus infection. *Cancer*.

[6] Kalmadi S., McNeill G., Davis M., Peereboom D., Adelstein D., Mekhail T. (2006). Phase II trial of weekly docetaxel and gemcitabine as first-line therapy for patients with advanced non-small cell lung cancer. *Medical Oncology*.

[7] Sasaki H., Yano M., Kawano O., Hikosaka Y., Fujii Y. (2012). Thymoma associated with fatal myocarditis and polymyositis in a 58-year-old man following treatment with carboplatin and paclitaxel: a case report. *Oncology Letters*.

[8] Winkelmann R. R., Yiannias J. A., Dicaudo D. J. (2014). Paclitaxel-induced diffuse cutaneous sclerosis: a case with associated esophageal dysmotility, Raynaud's phenomenon, and myositis. *The International Journal of Dermatology*.

[9] Perel-Winkler A. C., Derk C. T. (2014). Diffuse cutaneous mucinosis in dermatomyositis: a case report and review of the literature. *Case Reports in Dermatological Medicine*.

[10] Bachegowda L. S., Makower D. F., Sparano J. A. (2014). Taxanes: impact on breast cancer therapy. *Anti-Cancer Drugs*.

[11] Hilkens P. H. E., Verweij J., Stoter G., Vecht C. J., van Putten W. L. J., van den Bent M. J. (1996). Peripheral neurotoxicity induced by docetaxel. *Neurology*.

[12] Hilkens P. H. E., Pronk L. C., Verweij J., Vecht C. J., Van Putten W. L. J., Van Den Bent M. J. (1997). Peripheral neuropathy induced by combination chemotherapy of docetaxel and cisplatin. *British Journal of Cancer*.

[13] Wiernik P. H., Schwartz E. L., Strauman J. J., Dutcher J. P., Lipton R. B., Paietta E. (1987). Phase I clinical and pharmacokinetic study of taxol. *Cancer Research*.

[14] Donehower R. C., Rowinsky E. K., Grochow L. B., Longnecker S. M., Ettinger D. S. (1987). Phase I trial of taxol in patients with advanced cancer. *Cancer Treatment Reports*.

[15] Sulkes A., Beller U., Peretz T. (1994). Taxol: initial Israeli experience with a novel anticancer agent. *Israel Journal of Medical Sciences*.

[16] Valero V., Holmes F. A., Walters R. S. (1995). Phase II trial of docetaxel: a new, highly effective antineoplastic agent in the management of patients with anthracycline resistant metastatic breast cancer. *Journal of Clinical Oncology*.

[17] Giaccone G., Huizing M., Huinink W. T. B. (1994). Preliminary results of two dose-finding studies of paclitaxel (taxol) and carboplatin in non-small cell lung and ovarian cancer: a European Cancer Center effort. *Seminars in Oncology*.

[18] Rowinsky E. K., Burke P. J., Karp J. E., Tucker R. W., Ettinger D. S., Donehower R. C. (1989). Phase I and pharmacodynamics study of taxol in refractory acute leukemias. *Cancer Research*.

[19] Saibil S., Fitzgerald B., Freedman O. C. (2010). Incidence of taxane-induced pain and distress in patients receiving chemotherapy for early-stage breast cancer: a retrospective, outcomes-based survey. *Current Oncology*.

[20] Lipton R. B., Apfel S. C., Dutcher J. P. (1989). Taxol produces a predominantly sensory neuropathy. *Neurology*.

[21] Freilich R. J., Balmaceda C., Seidman A. D., Rubin M., DeAngelis L. M. (1996). Motor neuropathy due to docetaxel and paclitaxel. *Neurology*.

[22] Callen J. P. (1994). Myositis and malignancy. *Current Opinion in Rheumatology*.

[23] Zahr Z. A., Baer A. N. (2011). Malignancy in myositis. *Current Rheumatology Reports*.

[24] Koike H., Tanaka F., Sobue G. (2011). Paraneoplastic neuropathy: wide-ranging clinicopathological manifestations. *Current Opinion in Neurology*.

[25] Nakao Y., Yokoyama M., Nishiyama S. (2013). Pyomyositis associated with chemotherapy for endometrial cancer: a case report. *World Journal of Surgical Oncology*.

[26] Singh P., Chan W., Blomfield P., McIntosh R. (2010). Pyomyositis after chemotherapy for endometrial cancer. *International Journal of Gynecological Cancer*.

[27] Albers J. W., Pop-Busui R. (2014). Diabetic neuropathy: mechanisms, emerging treatments, and subtypes. *Current Neurology and Neuroscience Reports*.

[28] Dyck P. J. B., Windebank A. J. (2002). Diabetic and nondiabetic lumbosacral radiculoplexus neuropathies: new insights into pathophysiology and treatment. *Muscle & Nerve*.

[29] Choudhury B., Saikia U., Sarma D., Saikia M., Choudhury S. D., Bhuyan D. (2011). Diabetic myonecrosis: an underreported complication of diabetes mellitus. *Indian Journal of Endocrinology and Metabolism*.

[30] Horton W. B., Taylor J. S., Ragland T. J., Subauste A. R. (2015). Diabetic muscle infarction: a systematic review. *BMJ Open Diabetes Research & Care*.

[31] Schulze M., Kötter I., Ernemann U. (2009). MRI findings in inflammatory muscle diseases and their noninflammatory mimics. *American Journal of Roentgenology*.

[32] Klopstock T. (2008). Drug-induced myopathies. *Current Opinion in Neurology*.

[33] Pascuzzi R. M. (1998). Drugs and toxins associated with myopathies. *Current Opinion in Rheumatology*.

[34] Catapano A. L. (2012). Statin-induced myotoxicity: pharmacokinetic differences among statins and the risk of rhabdomyolysis, with particular reference to pitavastatin. *Current Vascular Pharmacology*.

[35] Thompson P. D., Clarkson P., Karas R. H. (2003). Statin-associated myopathy. *Journal of the American Medical Association*.

[36] Mohaupt M. G., Karas R. H., Babiychuk E. B. (2009). Association between statin-associated myopathy and skeletal muscle damage. *Canadian Medical Association Journal*.

[37] Rowinsky E. K., Chaudhry V., Forastiere A. A. (1993). Phase I and pharmacologic study of paclitaxel and cisplatin with granulocyte colony-stimulating factor: neuromuscular toxicity is dose-limiting. *Journal of Clinical Oncology*.

